# Anxiety status in outpatients of Type-2 diabetes mellitus

**DOI:** 10.12669/pjms.41.3.10487

**Published:** 2025-03

**Authors:** Azmat Ali Khan, Shaista Kanwal, Jamal Ahmad Khan, Azizul Hasan Aamir

**Affiliations:** 1 Azmat Ali Khan, MBBS, FCPS Medicine Department of Diabetes, Endocrinology and Metabolic Diseases, MTI Hayatabad Medical Complex, Peshawar, Pakistan; 2 Shaista Kanwal, MBBS, FCPS Medicine, FCPS Endocrinology, MRCP Department of Diabetes, Endocrinology and Metabolic Diseases, MTI Hayatabad Medical Complex, Peshawar, Pakistan; 3 Jamal Ahmad Khan, MBBS, FCPS Medicine Department of Diabetes, Endocrinology and Metabolic Diseases, MTI Hayatabad Medical Complex, Peshawar, Pakistan; 4 Azizul Hasan Aamir, MBBS, MRCP, FRCP, FACE, CCST. Department of Diabetes, Endocrinology and Metabolic Diseases, MTI Hayatabad Medical Complex, Peshawar, Pakistan

**Keywords:** Anxiety, Beck Anxiety Inventory, Type-2 DM

## Abstract

**Objectives:**

This study intended to determine the frequency of anxiety in outpatients of Type-2 diabetes mellitus (T2DM) and to evaluate its associated characteristics.

**Methods:**

This cross-sectional observational study was accomplished from July 1, 2023, to December 31, 2023, at the Department of Endocrinology at Hayatabad Medical Complex, Peshawar. The study comprised of individuals with T2DM who were at least 18 years of age and of either gender and who presented to the outpatient department for various reasons. The Beck Anxiety Inventory (BAI) was utilized to assess the respondents’ level of anxiety.

**Results:**

A total of 306 patients with T2DM were enrolled, of whom, 116 (37.9%) were males and 190 (62.1%) were females. The mean age and HbA1c of the participants were 50.7 ± 11.2 years and 12.02 ± 2.03%. Majority (40.2%) of the participants had moderate anxiety while low anxiety and severe level of anxiety were reported by 30.4% and 29.4% participants, respectively. There was a statistically significant association of anxiety with gender, education, employment, family history, comorbidities, and HbA1c. Educated patients (OR=2.7, 95% CI 1.5 to 5.01 p=0.001), and those with comorbidities (OR= 2.3, 95% CI 0.91 to 5.98, p=0.01) had higher odds for occurrence of anxiety.

**Conclusion:**

Almost 70% of the outpatients of T2DM had moderate to severe anxiety, with most of the clinicodemographic characteristics as potential associated factors. It is crucial for informing clinical practice, enhancing patient care, and promoting better mental health outcomes in this population.

## INTRODUCTION

Type-2 Diabetes mellitus (T2DM) is a multifaceted condition that has been linked to factors such as age, gender, ethnicity, educational attainment, anxiety and depression, sedentary lifestyles, and a family history of the condition.^1^ Over the past few decades, lower- and middle-income countries have seen greater increases in prevalence than higher-income countries.^2^ With one in four persons in the nation having diabetes, Pakistan has the third-highest number of diabetic patients (33 million) in the world, after China (140 million) and India (74 million).^3^

The bi-directional connection between T2DM and mental health conditions like depression and anxiety is well-documented.^4^ Anxiety and depression are estimated to affect 45-58% of T2DM outpatients in underdeveloped nations, which can be attributed to several factors like socio-economic challenges, limited access to healthcare resources, cultural stigmas surrounding mental health, and the burden of managing a chronic illness in resource-constrained settings. These psychosocial disorders are independently linked to increased mortality and morbidity.^5^ Conversely, they have been shown to exacerbate the negative health outcomes associated with DM, like impaired health related quality of life (HRQoL)^6^, non-adherence to medications, suboptimal glycaemic control, increased healthcare costs and increased risk of diabetes-related complications.^7^

Studies conducted in Pakistan have revealed that anxiety was reported by 66.6% of T2DM inpatients,^8^ and 50% of the T2DM outpatient population.^9^ Prior research from Pakistan and India has shown that compared to outpatients, hospitalized patients experienced much higher rates of anxiety.^8^,^10^ However, very little is known regarding the anxiety status of outpatient population of T2DM. It is critical to enhance the understanding of anxiety prevalence and associated factors among outpatients of T2DM. Therefore, the purpose of this study was to determine the frequency of anxiety in outpatients of T2DM and to study its associated characteristics. The study hypothesizes that outpatients with T2DM would have a greater prevalence of anxiety than the general population, and that clinical and demographic characteristics will be strongly correlated with the occurrence of these symptoms. This is imperative for informing clinical practice, enhancing patient care, and promoting better mental health outcomes in this population.

## METHODS

This cross-sectional observational study was carried at the Endocrinology department of the MTI-Hayatabad Medical Complex, Peshawar from July 1, 2023, to December 31, 2023. Nonprobability consecutive sampling was utilized to recruit patients. The OpenEpi program^11^ was employed for sample size calculation. The computed sample size was 306, while taking 95% confidence interval, a 5% margin of error, and a 27.4%^12^ prevalence of anxiety in patients with diabetes in Pakistan.

### Ethical Approval:

The study was approved by the hospital’s ethics committee (Approval Number# 1359, Dated: July 14, 2023).

The study comprised of individuals with T2DM who were at least 18 years of age and of either gender and who presented to the outpatient department for various reasons, such as glycaemic control, stable diabetic kidney disease, diabetic foot ulcers or infections (but not requiring admission), urinary tract infections (UTI), and routine follow up visits. Individuals with end-stage kidney disease, dementia, cerebrovascular accident, Type-I diabetes, gestational diabetes, patients with prior diagnosis of anxiety or depression or receiving treatment for that, patients with a history of death of close family member (spouse, sibling, parent, and child) in the previous six weeks, those who became jobless in the previous six weeks and those who refused to take part in the study were excluded. All research participants were guaranteed complete confidentiality of the data collected before conducting any interviews, and they gave their written consent after receiving all the information needed.

Demographic data was acquired, including age, sex, occupation, level of education, socioeconomic status, length of DM, comorbidities and complications, and therapy for DM. The Beck Anxiety Inventory (BAI) was used to assess the respondents’ level of anxiety.^13^ Patients were asked how much each of the 21 anxiety symptoms on the BAI has affected them over the course of the previous week. A rating scale was provided to each participant, with 0 denoting “not at all” and three denoting “severely, I could scarcely take it.”

A minimum of 0 and a maximum of 63 represent the overall score. The final score was calculated as the sum of the 21 components. The three groups for the overall anxiety score were severe levels of anxiety (score of ≥ 36), moderate levels of anxiety (score of 22–35), and low levels of anxiety (score of 0–21). Over a one-week period, the BAI has a good test-retest reliability (0.75) and a high internal consistency (Cronbachs α =.92).^13^

### Statistical Analysis:

Version 20 of SPSS was employed for the analysis. Biochemical parameters were presented as mean with standard deviation while frequency and percentages were presented for categorical variables. The study employed binary logistic regression analysis and the chi-square test to evaluate the association between the outcome variables (anxiety) and its related components. The logistic regression analysis demonstrated a substantial improvement in model fit relative to the null model (p < 0.001). The Cox and Snell R-square (0.184) and Nagelkerke R-square (0.260) demonstrated that the predictors accounted for a moderate proportion of the variability in anxiety status. The model’s overall prediction accuracy increased from 69.6% in Block 0 to 72.9% in Block One, indicating that the incorporation of predictors improved model performance. To evaluate significance, p has to be less than 0.05.

## RESULTS

A total of 306 patients with T2DM presenting to the outpatient department were enrolled in the study. Of these, 116 (37.9%) were males and 190 (62.1%) were females. The mean age and HbA1c of the study population was 50.7 ± 11.2 years and 12.02 ± 2.03%. Approximately, 58.2% (n=178) of the participants in the study were educated, 25.8% (n=79) were employed, and most of them belonged to either low (56.5%) or middle (42.5%) socioeconomic class. Almost 25.5% (n=78) of the participants had comorbidities. Approximately 23.5% of the participants had an HbA1c of < 10% whereas most (76.5%) of the participants had an HbA1c level of > 10%. Approximately, 33% of the participants had a duration of diabetes of more than 10 years. The clinical and demographic characteristics of the study cohort at baseline are presented in Table-I. With a maximum score of 60 and a lowest score of 0, the mean BAI score was 28.95 ± 11.9.

**Table-I:** Demographic and Clinical Characteristics of the Study Population at Baseline.

Patient Characteristic		Number of participants (n=306)	Percentage (%)
Gender	Male	116	37.9
	Female	190	62.1
Education	Uneducated	128	41.8
	Educated	178	58.2
Employment	Housewives	188	61.4
	Unemployed	39	12.7
	Employed	79	25.8
Socioeconomic Status	Low	173	56.5
	Middle	130	42.5
	High	3	1
Duration of DM	< 5 years	96	31.4
	5 - 10 years	109	35.6
	> 10 years	101	33
Family History	No	48	15.7
	Yes	258	84.3
Comorbidities	No	228	74.5
	Yes	78	25.5
HbA1c	6.5 - 8%	7	2.3
	8.1 - 10 %	65	21.2
	> 10 %	234	76.5
Anxiety	Low Anxiety	93	30.4
	Moderate Anxiety	123	40.2
	Severe Anxiety	90	29.4

Majority (40.2%) of the participants had moderate anxiety while low anxiety and severe levels of anxiety were reported by 30.4% and 29.4% participants, respectively. The association of the anxiety status with gender (p= < 0.001), education (p= < 0.001), and employment (p= < 0.001) status was statistically significant. Likewise, the association of anxiety status with family history of DM (p= 0.006), comorbidities (p=0.001) and HbA1c (p=0.004) was also found to be statistically significant. Whereas the association of anxiety status was not found to be statistically significant with socioeconomic status (p=0.2) and duration of DM (p=0.2). Women, educated patient population and those with HbA1c of more than 10% were more likely experience higher grades of anxiety. The association between the anxiety status in T2DM outpatients with various study variables is presented in Table-II.

**Table-II:** Association of Anxiety Status of Type 2 DM patients with various characteristics of the study population.

Patient Characteristics		Anxiety Status			Total (n=306)	p value

		Low (n=93)	Moderate (n=123)	Severe (n=90)		
Gender	Male	52	44	20	116	<0.001
	Female	41	79	70	190	
Education	Uneducated	51	57	20	128	<0.001
	Educated	42	66	70	178	
Employment	Housewives	41	79	68	188	<0.001
	Unemployed	12	16	11	39	
	Employed	40	28	11	79	
Socioeconomic status	Low	44	73	56	173	0.2
	Middle	47	49	34	130	
	High	2	1	0	3	
Duration of DM	< 6 years	35	31	30	96	0.2
	6 - 10 years	34	48	27	109	
	>10 years	24	44	33	101	
Family history	No	20	23	5	48	0.006
	Yes	73	100	85	258	
Comorbidities	No	81	91	56	228	0.001
	Yes	12	32	34	78	
	Less than 8	5	2	0	7	0.004
HbA1c (%)	8.1 to 10	28	17	20	65	
	More than 10	60	104	70	234	

Logistic regression analysis revealed that patients with an HbA1c of 8-10% (OR=2.4, 95% CI 0.4 to 15.2, p= 0.3) and HbA1c >10 % (OR=4.3, 95% CI 0.7 to 25.8 p= 0.1) were more likely to suffer from anxiety. Females (OR=3.1, 95% CI 0.7 to 13.6 p=0.14), educated patients (OR=2.7, 95% CI 1.5 to 5.01 p=0.001), low socioeconomic class (OR=8.5, 95% CI 0.6 to 119.3 p=0.1) and unemployed (OR=1.9, 95% CI 0.8 to 4.9 p= 0.2) had higher odds for occurrence of anxiety. Likewise, patients with a DM duration of more than 10 years (OR=1.06, 95% CI 0.5 to 2.2, p=0.9), those with a family history of DM (OR=3.9, 95% CI 1.7 to 9.1, p=0.4), and those with comorbidities (OR= 2.3, 95% CI 0.91 to 5.98, p=0.01) had higher odds for higher grades of anxiety. It was interesting to note that patients with HbA1c of 8.1 to 10% (OR= 2.3, 95% CI 0.4 to 15.2, p= 0.3) and those with HbA1c of more than 10% (OR=4.3, 95% CI 0.7 to 25.8, p=0.1) also had higher odds for occurrence of anxiety. These findings are displayed in Table-III.

**Table-III:** Logistic Regression Analysis of Various characteristics with Anxiety status.

Variable		Odds ratio	95% confidence interval	p value
Gender	Male	1		
	Female	3.1	0.7-13.6	0.14
Education	Uneducated	1		
	Educated	2.7	1.5-5.01	0.001
Employment	Employed	1		
	Housewives	1.4	0.3-6.5	0.7
	Unemployed	1.9	0.8-4.9	0.2
Socioeconomic status	High	1		
	Low	8.5	0.6-119.3	0.1
	Middle	5.5	0.4-74.7	0.2
Duration of DM	Less than 6 years	1		
	6-10 years	1.02	0.5-1.9	0.9
	More than 10 years	1.06	0.5-2.2	0.9
Family history	No	1		0.4
	Yes	3.9	1.7-9.1	
Comorbidities	No	1		0.01
	Yes	2.3	0.91-5.98	
HbA1c (%)	Less than 8	1		
	8 – 10	2.4	0.4-15.2	0.3
	More than 10	4.3	0.7-25.8	0.1

## DISCUSSION

According to our study’s results, most participants (40.2%) experienced moderate anxiety, while low anxiety and severe levels of anxiety were reported by 30.4% and 29.4% participants, respectively. Studies in Pakistan by Khwaja et al.^14^ and Azad et al.^9^ demonstrated an approximately 57.9% and 50% prevalence of anxiety in outpatient cohort of T2DM, respectively. This difference in the frequency of anxiety is due to utilization of different scales to measure the anxiety levels. Studies from India (17.3%)^15^ and Nepal (49.7%)^16^ have reported comparatively lower frequency of anxiety in this patient population. Another study conducted in 15 countries, comprising of adult patients with diabetes receiving outpatient treatment, revealed that the total prevalence of anxiety disorders among individuals with Type-2 diabetes was 18%; however, 2.8% of the research group exhibited multiple anxiety disorders. An elevated prevalence of anxiety disorders was noted in Ukraine, Saudi Arabia, and Argentina, whereas a diminished frequency was recorded in Bangladesh and India.^17^ Whereas 19.5% prevalence of anxiety was reported in T2DM population in USA.^18^

A possible explanation for the disparities in the occurrence of anxiety could be due to utilization of distinct inclusion criteria, sample sizes, recruiting techniques, screening instruments, and cut-off points used in these studies. The observed disparities among countries may be attributed to cultural variations in the reporting and experience of anxiety symptoms, potentially influenced by the stigma surrounding mental health issues or the robust social support provided by families in certain nations, which may facilitate the prevention of onset.^17^ Previous studies from Pakistan and India have demonstrated lower rates of anxiety in outpatients compared to inpatients of T2DM.^8^,^10^ Factors like controlled environment of outpatient, stronger support system, less intensive treatment, shorter stay in hospital, and reduced stigma contribute to the lower frequency of anxiety among outpatients compared to inpatients.^8^ Regarding severity of anxiety, most of the participants had moderate anxiety.

A study from Ecuadorian and Canadian outpatient cohort of T2DM also reported that most of the subjects had moderate to severe level of anxiety.^19^,^20^ Conversely, a study from Pakistan has reported a higher frequency of mild anxiety in this cohort.^8^ This variation in the findings could be due to the different anxiety scales used in these studies (Hospital Anxiety and Depression Scale (HADS) and Hamilton Anxiety Rating Scale (HAM-A) utilized in the former while BAI utilized in the present study).

Our study revealed that compared to men, women were 3.1 times more likely to experience anxiety. Similar studies from Pakistan and India have also reported a positive association between female gender and anxiety.^8^,^12^,^15^ A multinational study conducted in 15 countries also reported a higher prevalence of anxiety among female population.^17^ This high prevalence of anxiety problems in females with diabetes may mirror the general trend observed in anxiety disorders. Biological factors, social status deprivation, maladaptive coping methods, and the lack of a national support system for women are the possible reasons for this increased frequency of anxiety in women.^21^

Our study demonstrated that educated patients reported higher rates (OR=2.7, 95% CI 1.5 to 5.01, p= 0.001) of anxiety compared to those who were uneducated. This contrasts with other studies which reported higher rates of anxiety in uneducated patient population.^12^,^15^,^19^,^21^ It is essential to note that education itself is not a direct cause of anxiety. Rather, it’s the combination of individual traits, societal pressures, and life circumstances that can contribute to higher levels of anxiety among some educated individuals.^22^ Although education is frequently linked to improved disease management, it does not inherently correspond with reduced anxiety levels. Educated patients with Type-2 diabetes may have elevated levels of anxiety due to various interconnected factors, including heightened understanding of the disease’s dangers and complications, increased expectations for self-management, and enhanced involvement with healthcare systems. These elements may result in an increased sense of responsibility and apprehension for adverse outcomes, thereby contributing to heightened anxiety levels.^23^

The current study reported high rates of anxiety in patients with low socioeconomic status. Study by Sharif et al. revealed that people with low socioeconomic status were 4.2 times more likely to experience anxiety than people with stable socioeconomic status.^24^ Studies from Malysia and India have found a similar correlation.^15^,^25^ Factors like financial stress, unemployment, housing insecurity, limited social support and limited access to affordable mental health resources are the plausible reasons for this.^25^

The current study also demonstrated a strong association between anxiety and factors like poor glycaemic control, family history and comorbidities. It was evident that patients with an HbA1c of more than 10% were 4.3 (p = 0.1) times more likely to experience anxiety compared to those with levels between 6.5% and 8%. Study by Collins et al has demonstrated a significant association between poor glycemic control and anxiety stuats.^26^ Likewise, a positive association between comorbidities like hypertension, ischemic heart disease and poor glycaemic status with the anxiety have been reported by other studies as well.^12^,^15^,^19^,^25^ Suboptimal glycaemic control might intensify anxiety via physiological stress responses, psychological strain from diabetes management, fear of complications, and non-adherence to treatment. Variations in blood glucose levels can influence mental status, leading to worry and neglect, so worsening comorbidities. These pathways establish a complex interaction that exacerbates both anxiety and diabetes-related problems.^27^ These hjighlight the necessity for a comprehensive approach to diabetes management that include mental health assistance. Efficient treatment of anxiety may improve glycemic control and alleviate the psychological strain associated with chronic disease management.

Our study underscores the significant correlations between anxiety and several demographic and clinical characteristics, including gender, education, and glycemic control. Significantly, we noted that educated patients exhibited elevated anxiety levels, a novel finding in the current literature. This data indicates that heightened awareness and societal demands regarding well-being may exacerbate anxiety in educated adults with T2DM.

### Strengths:

The utilization of validated instruments to evaluate anxiety levels enhances the reliability of our findings. Our study’s extensive examination of demographic and clinical characteristics offers a nuanced perspective on the association between anxiety and T2DM.

### Limitations:

Firstly, anxiety state was measured using a psychometric scale instead of a structured interview, which makes it difficult to take into consideration the physiological components of mental symptoms. While psychometric scales are useful tools for assessing anxiety symptoms in large samples, they may not capture the full spectrum of mental health issues. Secondly, a causal relationship between diabetes and anxiety symptoms cannot be established as it was a cross-sectional study. Furthermore, selection bias and generalizability are critical factors to consider. The study’s findings, derived from outpatients in a particular clinical setting, may not accurately reflect the wider community of adults with T2DM. Consequently, the results may not be applicable to patients with T2DM who are not pursuing outpatient therapy or those with more advanced stages of the condition.

## CONCLUSION

Almost 70% of the outpatients of T2DM had moderate to severe anxiety, with most of the demographic and clinical characteristics as potential associated factors. These findings highlight the significance of identifying and managing anxiety in individuals with T2DM. Considering the substantial correlations between anxiety and elements like glycemic control and comorbidities, it is imperative to incorporate mental health care into the management of T2DM. Integrated care models that incorporate psychological support may enhance health outcomes, as addressing both physical and mental health dimensions of the disease is crucial for optimal patient welfare. Future interventions must emphasize the incorporation of mental health assessment and treatment within diabetes control programs.

### Future recommendations:

Longitudinal designs to investigate the temporal association between anxiety symptoms and diabetes and incorporating structured clinical interviews alongside psychometric scales may offer a more thorough comprehension of the intricate interactions between anxiety and diabetes. Subsequent research with a more heterogeneous sample, encompassing various healthcare environments or demographics, might enhance the generalizability of the findings. Future study should ought to investigate the causal link between anxiety and inadequate glycemic control, along with the possible advantages of integrated mental health interventions in enhancing diabetes outcomes.

### Authors’ contribution:

**AAK:** Conceived, designed, participated in data collection, literature review, statistical analysis & preparation of the manuscript.

**SK:** Did literature review, performed statistical analysis, interpreted the data, and helped in drafted. **JAK:** Did literature review, participated in data collection, statistical analysis and data interpretation, and helped in drafting the manuscript.

**AHA:** Conceived, designed, did literature review, statistical analysis and interpretation of data and critically revised the manuscript.

All authors gave final approval for publication of the manuscript and are responsible for the integrity of the study.
